# Suppression of mucin 2 promotes interleukin-6 secretion and tumor growth in an orthotopic immune-competent colon cancer animal model

**DOI:** 10.3892/or.2014.3544

**Published:** 2014-10-13

**Authors:** YAN-SHEN SHAN, HUI-PING HSU, MING-DERG LAI, MENG-CHI YEN, JUNG-HUA FANG, TZU-YANG WENG, YI-LING CHEN

**Affiliations:** 1 Department of Surgery, National Cheng Kung University Hospital, College of Medicine, National Cheng Kung University, Tainan, Taiwan, R.O.C.; 2 Department of Biochemistry and Molecular Biology, College of Medicine, National Cheng Kung University, Tainan, Taiwan, R.O.C.; 3 Department of Emergency Medicine, Kaohsiung Medical University Hospital, Kaohsiung Medical University, Kaohsiung, Taiwan, R.O.C.; 4 Laboratory Animal Center, College of Medicine, National Cheng Kung University, Tainan, Taiwan, R.O.C.; 5 Department of Senior Citizen Service Management, Chia Nan University of Pharmacy and Science, Tainan, Taiwan, R.O.C.

**Keywords:** colon cancer, interleukin-6, mucin 2, small interfering RNA, therapeutic effects

## Abstract

Mucin 2 (MUC2) is the major secreted mucin of the large intestine and is expressed by adenomas and mucinous carcinomas. Since colon cancer is associated with a proinflammatory microenvironment and dysregulated MUC2 expression, the aim of this study was to characterize the effects of *MUC2* gene expression in colon tumor progression using colonic cancer cells. CT26 colon cancer cells were stably transfected with MUC2 siRNA (MUC2 RNAi) or a control construct containing a nonspecific sequence (scrambled RNAi). Expression of MUC2 was significantly decreased in the MUC2 RNAi cell clones. Although MUC2 suppression did not affect the cell growth of colon cancer cells *in vitro*, MUC2 knockdown promoted tumor growth in an orthotopic colon cancer model *in vivo*. MUC2 silencing also increased interleukin (IL)-6 secretion by colon cancer cells. IL-6 neutralization attenuated tumor formation by MUC2 RNAi cells; it also increased CD8 T cell infiltration into the peritoneum. Taken together, to the best of our knowledge, this is the first study indicating that the immune response to cancer cells plays an important role in tumor growth regulated by MUC2. Furthermore, given the effects of MUC2 on IL-6 secretion, its targeting may represent a potentially useful strategy to treat colonic carcinomas.

## Introduction

Mucins are the major glycoproteins of the gastrointestinal tract; secretory mucin 2 (MUC2) is the main component of the protective mucus layer. Although MUC2 expression is decreased in colorectal adenocarcinoma, colonic mucinous carcinomas are characterized by overexpression or ectopic expression of MUC2 (1). In contrast, the suppressive effect of MUC2 in colorectal cancer (CRC) was demonstrated in MUC2-null mice (2), suggesting that it has a protective effect in the colon. Although MUC2 overexpression was specifically associated with mucinous carcinoma, its effects on tumor progression remain unclear.

Intestinal inflammation is a crucial component of tumor development and metastasis (3), and polymorphonuclear neutrophils and macrophages may affect tumor development and progression in CRC (3). The effects of interleukin (IL)-6 on tumor growth are multifaceted. In previous studies, tumor-infiltrating macrophages (TIMs) were found to express a high level of IL-6 (4,5), which is implicated in CRC carcinogenesis. In prostatic carcinoma, IL-6 acts as a growth factor (6,7). In CRC patients, IL-6 levels were significantly higher than these levels in healthy controls (8,9) and were correlated with tumor stage, tumor size, liver metastasis and poor survival (8,10–12). Moreover, macrophage secretion of IL-6 decreased MUC2 expression in a human colon cancer cell line through phosphorylation of STAT3 (13). IL-6 can also regulate the immune microenvironment surrounding the tumor (14). However, the effects of MUC2 expression on IL-6 secretion by colon cancer cells have not been determined. Therefore, we employed RNA interference (RNAi) to suppress MUC2 expression in mouse colon carcinoma cells, and evaluated the effects of MUC2 suppression on IL-6 production and tumor growth.

## Materials and methods

### Cell culture

CT26 colon carcinoma cells (BALB/c mouse origin) were kindly provided by Dr M.D. Lai of the National Cheng Kung University (Tainan, Taiwan). CT26 cells were maintained in high glucose Dulbecco’s modified Eagle’s medium (DMEM) (Gibco-Invitrogen) containing 10% FBS (HyClone Laboratories, Logan, UT, USA) and 1% penicillin/streptomycin. All cell lines were stored in a humidified 37°C incubator with 5% CO_2_.

### Mice

Eight-week-old BALB/c (H_2_^d^) mice and non-obese diabetes/severe combined immunodeficiency (NOD/SCID) mice, NOD.CB17-PRKDC, were purchased from the Laboratory Animal Center of National Cheng Kung University and were maintained under pathogen-free conditions. All animal experiments were conducted with the approval of the Institutional Animal Care and Use Committee (IACUC) of the National Cheng Kung University.

### RNA interference

siRNA targeting mouse MUC2 was constructed within the pHsU6 vector as described previously (15) using the following target sequences: 5′-GCTATGT GCCTGGCTCTAA-3′ (RNAi-1), 5′-GCAACAAGTGCACC TT CTT-3′ (RNAi-2) and 5′-GCTGCCCTACAAACTGTTT-3′ (RNAi-3). Cells were cotransfected with the pHsU6 vectors containing the shRNA target sequences, a nonspecific sequence (scrambled shRNA), and pCMV-neo using Lipofectamine 2000 transfection reagent (Invitrogen, Carlsbad, CA, USA). The stable transfectants were selected with G418 (Calbiochem, San Diego, CA, USA). We established three clones of MUC2-suppressed cells, including CT26 MUC2 RNAi-1, CT26 MUC2 RNAi-2 and CT26 MUC2 RNAi-3. The clonal cell lines were maintained in complete medium with 250 μg/ml G418. To monitor the efficacy of MUC2 silencing, the expression of MUC2 in CT26 stable transfectants was analyzed by western blotting.

### Western blot analysis

A MUC2-specific antibody was obtained by inoculating rabbits with the mouse MUC2 peptide, CVRTRRSSPRFLGRK (c-terminal position 911–924). Peptides used in this study were synthesized and purified by Genemed Synthesis (San Antonio, TX, USA). Total cell lysates were prepared and analyzed by SDS-PAGE as previously described (16). Immunodetection was performed using a horse-radish peroxidase (HRP)-based SuperSignal Chemiluminescent Substrate (Pierce, Rockford, IL, USA). For quantification, the bands were measured by the AlphaImager 2200 system (Alpha Innotech, San Leandro, CA, USA) and normalized by the density obtained for β-actin.

### Cell proliferation assay

Cells (1×10^3^/well) were seeded in quadruplicate onto 96-well plates and incubated at 37°C under 5% CO_2_. At 24, 48 and 72 h, viable cell numbers were measured using the CellTiter 96 Aqueous One Solution Cell Proliferation assay (Promega, Madison, WI, USA) according to the manufacturer’s instructions. The proliferation curves were constructed by calculating the mean value of absorbance at 490 nm with an Ultra Multifunctional Microplate Reader (Tecan, Durham, NC, USA).

### Orthotopic model of colon adenocarcinoma

Eight-week-old female BALB/c mice were anesthetized by intraperitoneal injection of Zoletil (50 mg/kg; Parnell Laboratories, Alexandria, NSW, Australia) and xylazine (10 mg/kg; Troy Laboratories, Glendenning, NSW, Australia). After making a small median abdominal incision in the mice under anesthesia, cecums were exteriorized and 1×10^6^ cells (scramble RNA, MUC2 RNAi-1, RNAi-2 or RNAi-3 tumor cells) in 0.05 ml of PBS were injected into the middle wall of the greater curvature of the cecal wall using a 1-cc U-100 insulin disposable syringe (Becton-Dickinson, Franklin Lakes, NJ, USA). Bipolar (ICC, ER BE) coagulation was used when the needle was removed from the wall of the cecum. Each cecum was then returned to the peritoneal cavity, and the abdominal wall and skin were closed with a Dexon 4-0 surgical suture (Ethicon, Bridgewater, NJ, USA). The mice were sacrificed 11–17 days after the tumor cell implantation or when moribund. The whole cecums from mice were excised, washed, removed of remaining diet, and weighed. For IL-6 antibody neutralization, BALB/c mice were pretreated with an injection of 100 μg/mouse of IL6-neutralizing antibody or isotype control antibody (both from BD Pharmingen, San Diego, CA, USA) 2 days prior to tumor cell injection. After tumor cell injection, mice were treated with IL-6 neutralizing antibody or control antibody every 3 days by intraperitoneal injection as previously described (17).

### Cytokine array and enzyme-linked immunosorbent assay (ELISA)

To collect culture supernatants, scrambled control and CT26 MUC2 RNAi-1 cells were cultured for 48 h in serum-free medium after which the supernatants were collected and measured using Mouse Cytokine Antibody Array II kit (RayBiotech, Norcross, GA, USA) and an IL-6 ELISA (R&D Systems, Minneapolis, MN, USA) according to the manufacturers’ instructions. The signal intensity of the array was scanned and quantified by densitometry using an AlphaImager 2200 system (Alpha Innotech) and normalized to the positive control.

### Flow cytometric analysis

To characterize the immune cells of the peritoneal fluid *in vivo*, peritoneal fluid was isolated and subjected to flow cytometry as previously described (16,18). The following fluorescent-labeled antibodies were purchased from BD Bioscience: rat anti-Ly6G-FITC, anti-CD4-PE, anti-CD8-PE, anti-CD11b-PE and isotype controls (FITC and PE). Data analysis was evaluated using a flow cytometer (FACScan; Becton-Dickinson).

### Statistical analysis

Data are expressed as means ± SD. Statistical analyses were performed using the Student’s t-test. A P-value <0.05 was considered significant for all comparisons.

## Results

### Suppression of MUC2 expression in mouse CT26 colon carcinoma cells by RNAi

**Figure 1:**
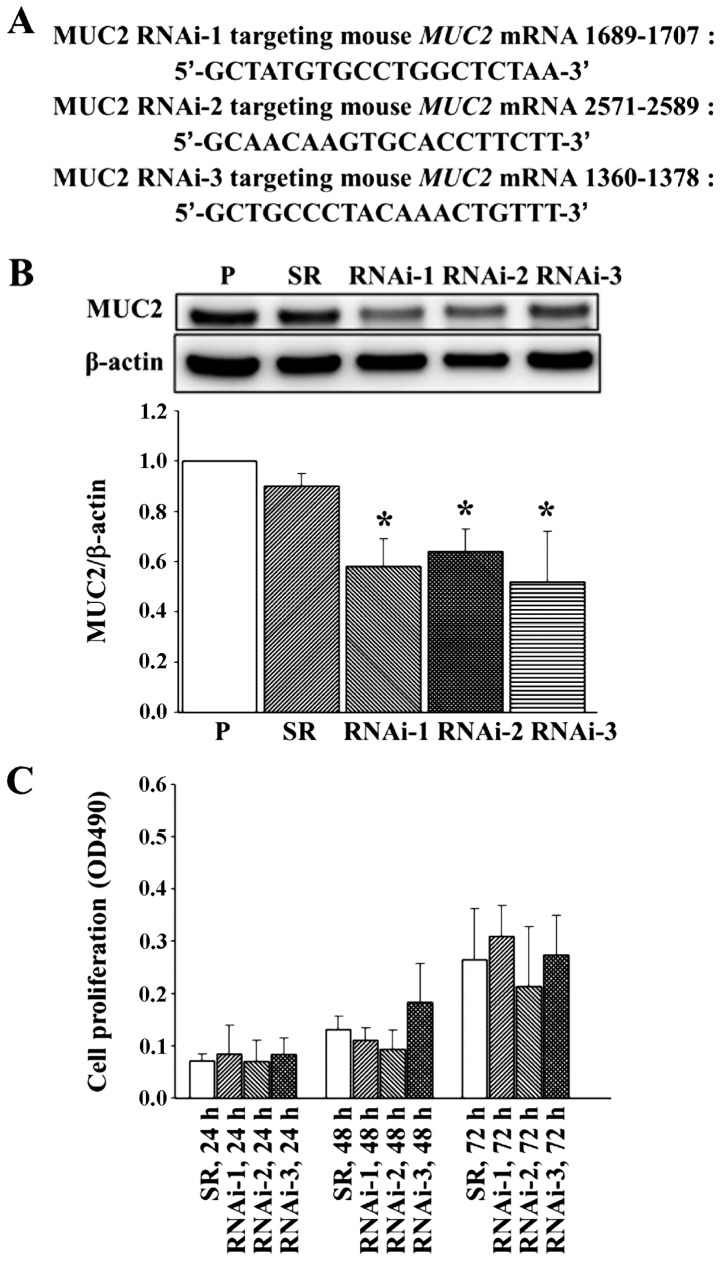
Mucin 2 (MUC2)-specific shRNA decreases MUC2 protein expression in CT26 cell clones. (A) RNAi targets on the coding sequence of MUC2. (B) MUC2-specific shRNA decreased MUC2 protein expression in the MUC2 RNAi-derived cell clones as detected by western blot analysis. β-actin was used as an internal control. ^*^P<0.05, when compared to a nonspecific sequence scrambled RNA (SR). P, parental cells; RNAi-1, RNAi-2 and RNAi-3, MUC2-specific shRNA-expressing cells. Results were obtained from three independent experiments. (C) MUC2 silencing in CT26 cell clones did not inhibit cell proliferation *in vitro*. The mean value ± SD of absorbance at 490 nm is shown for three independent experiments.

To study the role of MUC2 in colon cancer, we used shRNA to suppress the expression of MUC2 in the CT26 murine colon cancer cell line, which expresses high levels of MUC2. Three shRNAs targeting different sites on MUC2 were transfected into CT26 cells, and the stable transfectants were established by G418 selection. The vector control was established by transfecting CT26 cells with an empty vector, and the scrambled RNA (SR) transfectant contains a different shRNA sequence with the same base composition of RNAi-1. Stable cells expressing three RNAi target sequences were established: MUC2 RNAi-1, MUC2 RNAi-2 and MUC2 RNAi-3 (Fig. 1A). Western blot analysis confirmed that the SR did not alter MUC2 protein levels (Fig. 1B). However, MUC2 expression was significantly decreased in the MUC2 RNAi-1, MUC2 RNAi-2 and MUC2 RNAi-3 CT26 cells (Fig. 1B).

### MUC2 suppression does not affect cell proliferation in vitro

To determine the effects of MUC2 suppression on cell growth, cells were seeded at a low density, and growth rates were determined for the parental cell line and its derived cell clones at 24, 48 and 72 h. MUC2 silencing did not inhibit the *in vitro* proliferation of CT26 cells (Fig. 1C).

### Effect of MUC2 suppression on CT26 tumor growth in vivo

**Figure 2:**
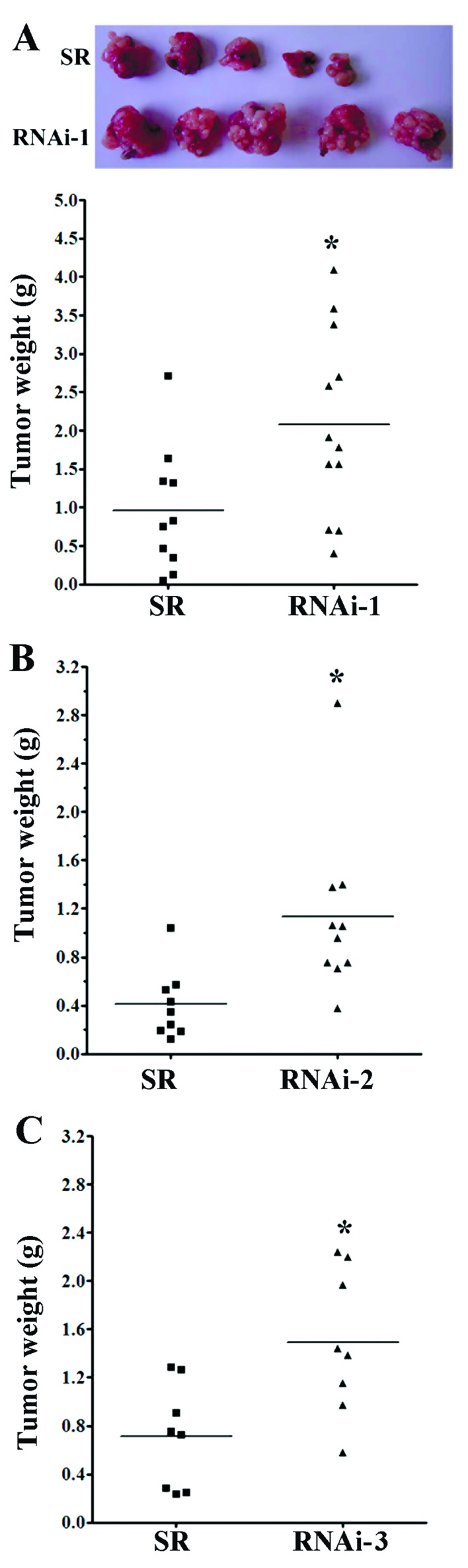
Mucin 2 (MUC2) silencing in CT26 cell clones enhances tumorigenicity *in vivo*. (A) The macroscopic appearance of the tumor masses after the orthotopic injection of scramble RNA (SR) and MUC2 RNAi-1 cell clones. The tumor weights of (A) MUC2 RNAi-1, (B) MUC2 RNAi-2 and (C) MUC2 RNAi-3 tumor-bearing mice were significantly increased on day 17 as compared to the tumor weights of the SR tumor-bearing mice. ^*^P<0.05 compared to SR mice. Results are expressed as the mean tumor weight, and the data are shown for two independent experiments.

To investigate the role of MUC2 in a native tumor environment, we examined the effects of MUC2 knockdown in an orthotopic immune-competent animal model. One million SR control or MUC2 shRNA-expressing cells were implanted orthotopically in BALB/c mice, and macroscopic tumor nodules indicative of tumor formation were detected (Fig. 2A). The tumor weight of mice injected with SR cells was significantly lower than the tumor weights of the mice injected with RNAi-1 (Fig. 2A), RNAi-2 (Fig. 2B) and RNAi-3 cells (Fig. 2C) at day 17. These results demonstrated that knockdown of MUC2 promoted the tumor growth of colon cancer cells *in vivo*, suggesting that MUC2 plays an important role in the tumorigenicity of colon cancer.

**Figure 3:**
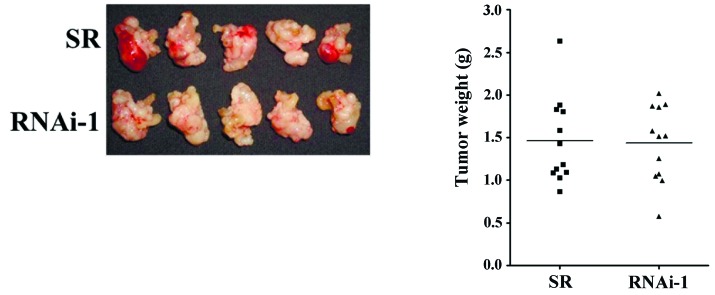
*In vivo* orthotopic growth of CT26-scramble RNA (SR) and CT26 mucin 2 (MUC2) RNAi-1 tumors in non-obese diabetes/severe combined immunodeficiency (NOD/SCID) mice. The macroscopic appearance of the tumor masses after the orthotopic injection of the SR and MUC2 RNAi-1 cell clones into NOD/SCID mice.

Since the growth of the MUC2 shRNA transfectants was not altered *in vitro*, we hypothesized that the changes in tumor growth *in vivo* may result from altered tumor-microenvironment interaction. To assess the potential immunological effects of MUC2 on tumor progression, tumor growth was measured in the immune-deficient NOD/SCID mice. In the absence of NK cells, macrophages, B and T cells, the mean tumor mass of mice implanted with the SR cells was similar to the mean tumor mass of mice implanted with MUC2 RNAi-1 cells (Fig. 3), suggesting that the effects of MUC2 were dependent upon the presence of a competent immune system.

### Suppression of MUC2 increases IL-6 secretion by CT26 colon cancer cells

**Figure 4:**
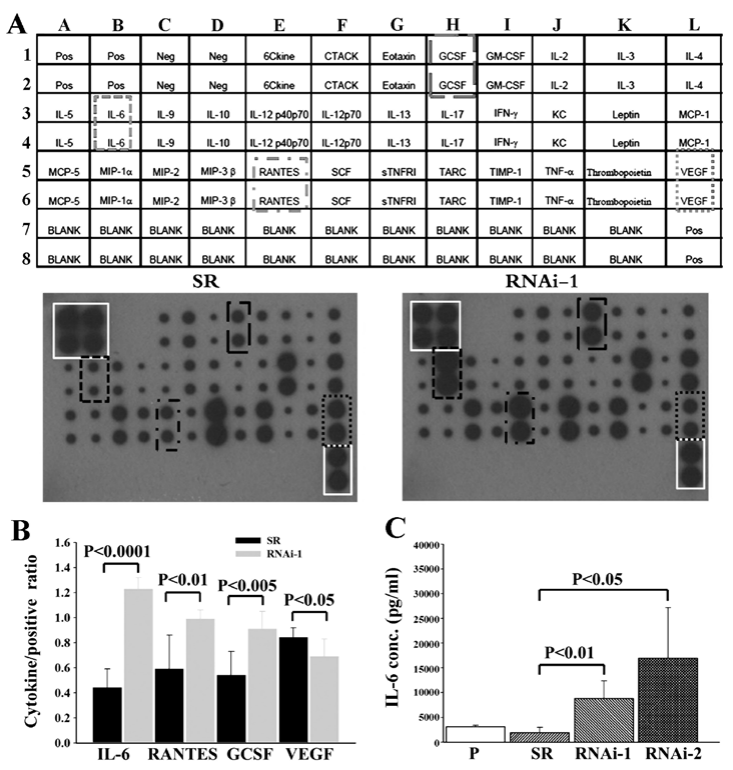
Cytokine secretion into condition medium after mucin 2 (MUC2) silencing in CT26 cell clones. (A) Cytokine array of conditioned media from scrambled RNA (SR) control (left panel) and MUC2 RNAi-1 cells (right panel) after 48 h in culture. (B) Cytokine assay of the conditioned media shows that the secretion of interleukin-6 (IL-6), regulated on activation, normal T cell expressed and secreted (RANTES) and granulocyte colony-stimulating factor (GCSF) was increased while secretion of vascular endothelial growth factor (VEGF) was decreased in the RNAi-1 group as compared with the SR group. Data are shown for three independent experiments. (C) Conditioned medium from CT26 parental cells, SR, MUC2 RNAi-1 and MUC2 RNAi-2 cells was analyzed for IL-6 secretion using ELISA. Results are expressed as the mean pg/ml ± SD, and the data are shown for three independent experiments.

Given the importance of a functional immune system for the effects of MUC2 on tumor growth, we further investigated whether cancer cell-secreted cytokines were involved in the tumor microenvironment. Specifically, the cytokine profile consisting of 32 different factors in the conditioned medium of SR and MUC2 RNAi-1 cells was compared 48 h after serum-deprivation. Conditioned medium from MUC2 RNAi-1 cells had significantly increased IL-6, regulated on activation, normal T cell expressed and secreted (RANTES) and granulocyte colony-stimulating factor (GCSF) expression and decreased vascular endothelial growth factor (VEGF) expression compared to the SR conditioned medium (Fig. 4). IL-6 secretion by SR and MUC2 RNAi-1 cells 48 h after serum-deprivation was further quantified by ELISA. MUC2 RNAi-1 and MUC2 RNAi-2 cells secreted significantly higher levels of IL-6 than SR cells (Fig. 4C). Therefore, MUC2 expression by colon cancer cells alters IL-6 secretion.

### IL-6 neutralization attenuates tumor formation by CT26 MUC2 knockdown cells

**Figure 5:**
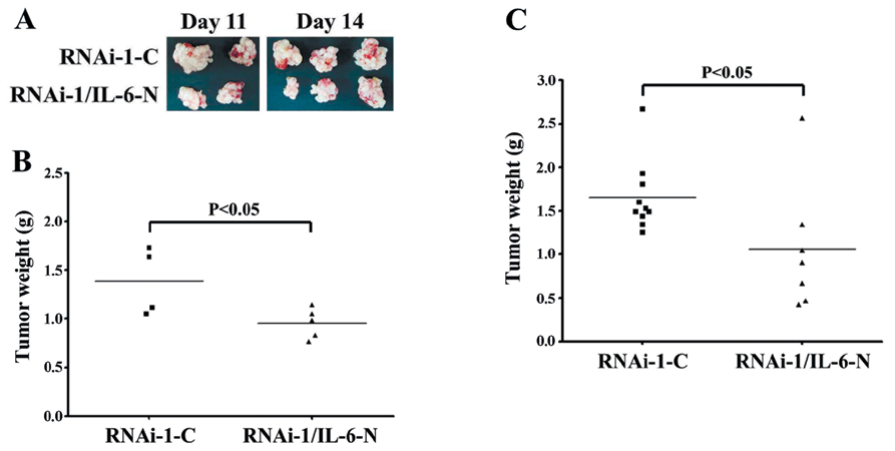
Mucin 2 (MUC2)-knockdown tumor growth was retarded with interleukin-6 (IL-6) neutralizing antibody treatment. (A) The macroscopic appearance of the tumor masses after the orthotopic injection of MUC2 RNAi-1 cell clones in mice injected every 3 days with 100 μg of either the IgG1 control antibody (RNAi-1-C) or the IL-6 neutralizing antibody (RNAi-1/IL-6-N). The mice were sacrificed (B) 11 days or (C) 14 days after the tumor cell implantation and tumor weight was determined.

To confirm the biological effect of IL-6 *in vivo*, we tested whether an IL-6 neutralization antibody could inhibit tumorigenic growth. Mice pretreated with either an IL-6-neutralizing antibody or control IgG antibody were injected with MUC2 RNAi-1 cells, and then treated every 3 days with either IL-6 or control antibody. The growth of MUC2 RNAi-1 tumors was reduced with the IL-6 neutralizing antibodies on day 11 (Fig. 5A and B) and day 14 (Fig. 5A and C). These experiments suggest that MUC2 knockdown enhanced IL-6 secretion and promoted tumor growth.

### IL-6 neutralization enhances the CD8-mediated immune response in CT26 cells after MUC2 silencing

**Figure 6:**
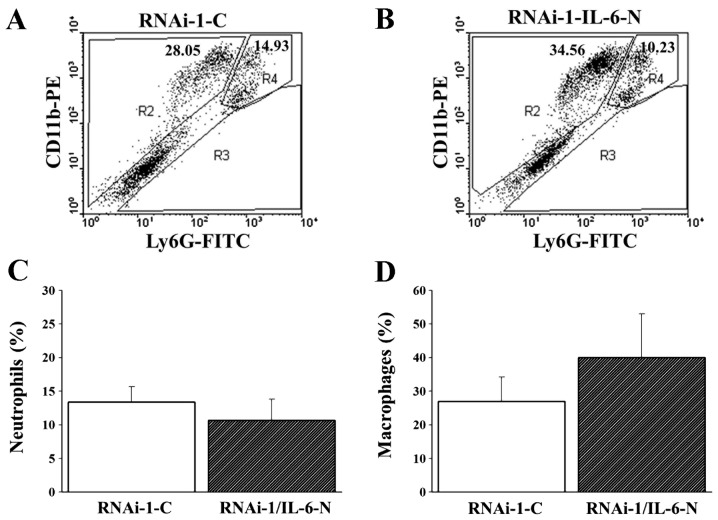
Ly6G^+^CD11b^+^ and Ly6G^−^CD11b^+^ cells were evaluated in the peritoneal fluid of RNAi-1-C and RNAi-1/interleukin-6 (IL-6)-N mice. Flow cytometry was performed on the peritoneal fluid from (A) RNAi-1-C and (B) RNAi-1/IL-6-N mice. The numbers shown are the percentage of total cells. R2, Ly6G^−^CD11b^+^ macrophages; R4, Ly6G^+^CD11b^+^ neutrophils; IgG1 control antibody, RNAi-1-C; IL6-neutralizing antibody, RNAi-1/IL-6-N. The percentages of (C) neutrophils (Ly6G^+^CD11b^+^) and (D) macrophages (Ly6G^−^CD11b^+^) isolated from the peritoneal fluid of RNAi-1-C and RNAi-1/IL-6-N mice were analyzed by flow cytometry 14 days after the tumor cell implantation.

**Figure 7:**
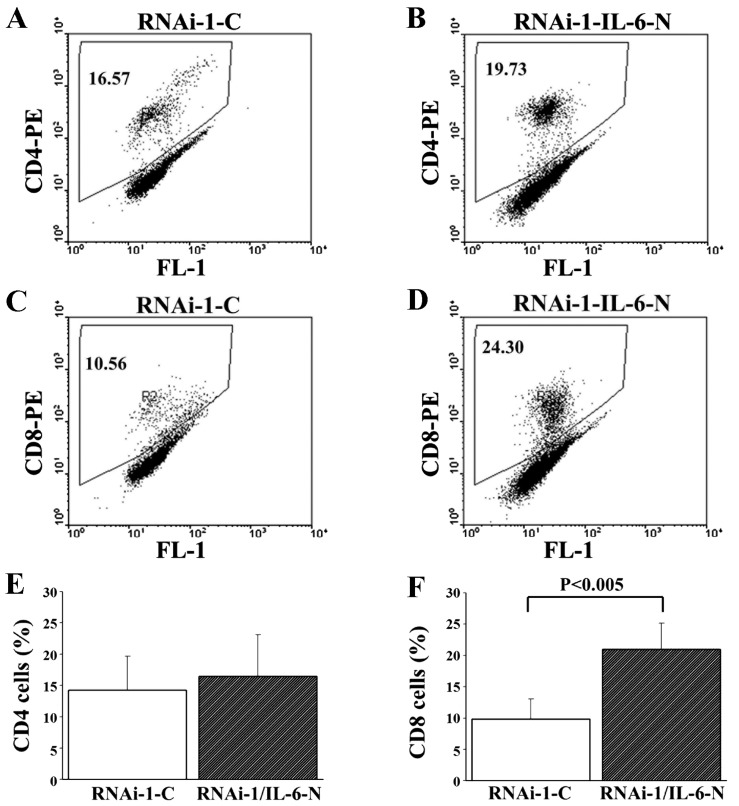
Interleukin-6 (IL-6) neutralization increases the proportion of CD8 T cells in the peritoneal fluid of mice bearing mucin 2 (MUC2) RNAi-1 tumors. MUC2 RNAi-1 tumor-bearing mice were injected every 3 days with 100 μg of either the IgG1 control antibody (RNAi-1-C) or the IL-6 neutralizing antibody (RNAi-1/IL-6-N). CD4 and CD8 T cells were evaluated in the peritoneal fluid of RNAi-1-C and RNAi-1/IL-6-N mice. Flow cytometry was performed on the peritoneal fluid from (A and C) RNAi-1-C and (B and D) RNAi-1/IL-6-N mice. The numbers shown are the percentage of total cells. IgG1 control antibody, RNAi-1-C; IL6-neutralizing antibody, RNAi-1/IL-6-N. The percentages of (E) CD4 and (F) CD8 T cells isolated from the peritoneal fluid of RNAi-1-C and RNAi-1/IL-6-N mice were analyzed by flow cytometry 14 days after tumor cell implantation.

To elucidate the possibility that MUC2 regulates IL-6 secretion and induces a local immune response in colon cancer cells, we analyzed Ly6G^+^CD11b^+^ neutrophil, Ly6G^−^CD11b^+^ macrophage, CD4 T cell and CD8 T cell levels in the peritoneal fluid of mice with MUC2 RNAi-1 cell tumors treated with control or IL-6 neutralization antibodies. Although there was no significant difference in the proportion of Ly6G^+^CD11b^+^ neutrophils, Ly6G^−^CD11b^+^ macrophages and CD4 T cells in the peritoneal fluid between the two groups on day 14 (Figs. 6 and 7A, B and E), the peritoneal fluid of the MUC2 RNAi-1 tumor-bearing mice treated with IL6-neutralizing antibody had a significantly greater proportion of CD8 T cells than MUC2 RNAi-1 tumor-bearing mice treated with the control antibody on day 14 (Fig. 7C, D and F). Thus, an IL-6 neutralizing antibody could inhibit the *in vivo* growth of MUC2 knockdown tumors, increasing CD8 T cell influx in the peritoneal cavity.

## Discussion

In the present study, we examined the effects of MUC2 on tumor cell growth, IL-6 secretion and the immune response in colon cancer. To the best of our knowledge, this is the first study to demonstrate that downregulation of MUC2 expression enhances IL-6 secretion as well as tumor growth. Thus, MUC2 had a protective effect during tumorigenesis. This observation is consistent with previous studies in which loss of MUC2 expression was associated with progression and metastasis in CRC (19–23). Moreover, CRC patients with a high MUC2/carcinoembryonic antigen (CEA) mRNA ratio in their lymph nodes had a significantly better prognosis than those with a low ratio (24). In addition, MUC2-positive CRC was found to be correlated with reduced disease recurrence and prolonged survival as well as a low incidence of liver and nodal metastasis (1,25,26).

In previous studies, MUC2 suppressed inflammation in the intestinal tract and inhibited intestinal tumorigenesis (2,27). In the present study, the secretion of IL-6, RANTES and GCSF was increased in the MUC2-knockdown tumor cells in comparison to the SR tumor cells. Both IL-6 and RANTES were associated with tumor progression, metastasis and macrophage activation in previous studies (28–32). In particular, IL-6 is an inflammatory cytokine released by T cells, macrophages and several cancer cell types (6,7,33). IL-6 production by tumor cells may act as an autocrine and/or paracrine growth factor during carcinogenesis, and various studies have proposed promising targets for CRC therapy using anti-IL-6 and anti-IL-6R antibodies as well as soluble gp130Fc (sgp130Fc) and selective small-molecule JAK inhibitors that suppress the IL-6/STAT3 pathway (34). The increased secretion of IL-6 by MUC2-knockdown tumor cells and enhanced tumor growth observed in the present study suggest that the protective mechanisms of MUC2 in colon cancer may be associated with its effects on suppressing the IL-6/STAT3 signaling pathway.

Inflammatory cells of the innate and adaptive immune system may affect different stages of tumor progression or metastasis in colon cancer. Since MUC2 silencing increased IL-6 secretion, we analyzed neutrophil, macrophage, CD4 T cell and CD8 T cell levels in the peritoneal fluid of mice. In this study, mice bearing MUC2-knockdown tumors and treated with IL-6 neutralizing antibodies displayed increased CD8 T cell infiltration into the peritoneal fluid. To the best of our knowledge, this is the first study to demonstrate that the immunoregulatory effects of MUC2 may represent a novel therapeutic method for treating cancer. Alternatively, tumor-associated antigens (TAAs) have served as a target for CTL immunotherapy as DNA vaccines have a therapeutic effect on established tumors through activation of a CD8 T cell-dependent pathway (35,36). Thus, it is intriguing that MUC2 may induce a systemic antitumor immune response and may have therapeutic value in the treatment of IL-6-secreting cancers. In conclusion, the therapeutic effects of MUC2 may be mediated at least in part by decreasing IL-6 secretion, inhibiting tumorigenicity, and inducing CD8 T cells *in vivo*.
